# Comparison of Fitness Cost and Virulence in Chromosome- and Plasmid-Mediated Colistin-Resistant *Escherichia coli*

**DOI:** 10.3389/fmicb.2020.00798

**Published:** 2020-05-13

**Authors:** Yujin Choi, Ji-Young Lee, Haejeong Lee, Myungseo Park, KyeongJin Kang, Suk-Kyung Lim, Dongwoo Shin, Kwan Soo Ko

**Affiliations:** ^1^Department of Microbiology, Sungkyunkwan University School of Medicine, Suwon, South Korea; ^2^Division of Antimicrobial Resistance, Korea Centers for Disease Control and Prevention, Cheongju, South Korea; ^3^Department of Integrative Biotechnology, College of Biotechnology and Bioengineering, Sungkyunkwan University, Suwon, South Korea; ^4^Department of Anatomy and Cell Biology, Sungkyunkwan University School of Medicine, Suwon, South Korea; ^5^Bacterial Disease Division, Animal and Plant Quarantine Agency, Gimcheon, South Korea

**Keywords:** colistin, *mcr-1*, fitness, antibiotic resistance, *Escherichia coli*

## Abstract

Five types of *Escherichia coli* strains were obtained and sequenced: colistin-susceptible (CL-S) strains, *in vitro* induced colistin-resistant (CL-IR) strains, *mcr-1-*negative colistin-resistant strains from livestock (CL-chrR), *mcr-1*-positive colistin-resistant strains (CL-mcrR), and *mcr-1*-transferred transconjugants (TC-mcr). Amino acid alterations of PmrAB, PhoPQ, and EptA were identified, and their mRNA expression was measured. Their growth rate was evaluated, and an *in vitro* competition assay was performed. Virulence was compared through serum resistance and survival in macrophages and *Drosophila melanogaster*. CL-IR and CL-chrR strains were colistin-resistant due to amino acid alterations in PmrAB, PhoPQ, or EptA, and their overexpression. All colistin-resistant strains did not show reduced growth rates compared with CL-S strains. CL-IR and CL-chrR strains were less competitive than the susceptible strain, but CL-mcrR strains were not. In addition, TC-mcr strains were also significantly more competitive than their respective parental susceptible strain. CL-IR strains had similar or decreased survival rates in human serum, macrophages, and fruit flies, compared with their parental, susceptible strains. CL-chrR strains were also less virulent than CL-S strains. Although CL-mcrR strains showed similar survival rates in human serum and fruit fly to CL-S strains, the survival rates of TC-mcr strains decreased significantly in human serum, macrophages, and fruit flies, compared with their susceptible recipient strain (J53). Chromosome-mediated, colistin-resistant *E. coli* strains have a fitness cost, but plasmids bearing *mcr-1* do not increase the fitness burden of *E. coli*. Along with high usage of polymyxins, the no fitness cost of *mcr-1*-positive strains may facilitate rapid spread of colistin resistance.

## Introduction

Colistin is considered as a “last resort” antibiotic against multi-drug resistant Gram-negative bacteria, despite its nephrotoxicity and neurotoxicity (Landman et al., 2008). After re-introduction in clinical settings, however, colistin-resistant bacteria are gradually emerging worldwide (Poirel et al., 2017). The well-known resistance mechanism to colistin is mutations in two-component regulatory systems, PmrAB and PhoPQ (Poirel et al., 2017). In *Escherichia coli*, PmrAB activates *arnBCADTEF* operon and *eptA*, and PhoPQ inhibits *eptB* by activating MgrR. *PmrD*, which is activated by PhoPQ, is known to stimulate the PmrAB system (Jeannot et al., 2017). The *arnBCADTEF* operon triggers the synthesis and addition of 4-amino-4-deoxy-L-arabinose (L-Ara4N) to lipid A, and *eptA* and *eptB* are involved in the synthesis and addition of phosphoethanolamine (PEtN). Mutations in PmrAB and PhoPQ result in the modification of lipid A and less anionic lipopolysaccharide (LPS), reducing the binding affinity to colistin (Jeannot et al., 2017).

Recently, the mobile colistin resistance gene, *mcr-1*, located on plasmids was first described in China (Liu et al., 2016). The existence of transmissible colistin resistance genes indicates the possibility of colistin resistance distribution through horizontal gene transfer and poses a crisis for public health care. Since the first report, numerous studies have demonstrated the spread of *mcr-1* and its variants across more than 40 countries on five continents at the time of writing (Sun et al., 2018). MCR-1 functions as PEtN transferase and adds PEtN to lipid A, leading to colistin resistance (Hinchliffe et al., 2018).

Generally, it is known that antibiotic resistance by acquisition of mobile elements, including plasmids, imposes fitness costs on the bacterial host (Andersson, 2006). However, it has also been described that the fitness burden is strongly dependent on the plasmid backbone and on the host (Humphrey et al., 2012). A fitness cost associated with colistin resistance due to chromosomal mutations has been reported mainly in *Acinetobacter baumannii* (López-Rojas et al., 2011; Pournaras et al., 2014). Although an *in vitro* and *in vivo* fitness cost of *mcr-1*-mediated colistin resistance have also been shown, some inconsistent results on fitness have been reported. While Zhang et al. (2017) and Nang et al. (2018) reported decreased fitness in *E. coli* and *Klebsiella pneumoniae* harboring *mcr-1*, respectively, a study by Tietgen et al. (2018) reported that plasmid-borne *mcr-1* did not reduce the fitness in *E. coli* but impaired it in *K. pneumoniae*. Meanwhile, stable maintenance and a large compensation of cost after passage of *mcr-1*-bearing *E. coli* has also been shown (Ma et al., 2018).

In this study, we compared the fitness and virulence of colistin-resistant *E. coli* strains caused by *mcr-1* in plasmids and mutations in chromosomal genes, using *in vitro* competition assays, serum resistance assays, bacterial infections in fruit flies, and bacterial survival assays in macrophages to understand what bacterial features can be affected by colistin resistance.

## Materials and Methods

### Bacterial Strains and Genotyping

Five types of *E. coli* strains were investigated in this study (Table 1). Three colistin-susceptible *E. coli* clinical strains (E015, E139, and E154), which are designated CL-S, were used for comparison and *in vitro* induction of colistin resistance. They were isolated from the blood of patients in South Korea. Colistin-resistant mutants (E015R, E139R, and E154R), which are briefly designated CL-IR, were obtained from the CL-S strains, respectively. Four colistin-resistant *E. coli* strains (QIA18, QIA24, QIA32, and QIA33), which were obtained from the Animal and Plant Quarantine Agency of Korea, were also included in this study. Their colistin resistance was not mediated by *mcr-1*, and they were designated CL-chrR. Three *mcr-1*-bearing colistin-resistant *E. coli* strains (EC006, EC019, and EC111) (designated CL-mcrR), which were isolated from livestock and have been reported previously (Lee et al., 2018), were included. Finally, the *mcr-1*-bearing plasmid of CL-mcrR strains were transconjugated into *E. coli* J53 strain. The *mcr-1*-transferred transconjugants (J53_pEC__006_, J53_pEC__019_, and J53_pEC__111_), designated TC-mcr, were included in this study.

**TABLE 1:** Bacterial strains used in this study.

*E. coli* strain	Description	Colistin MIC (mg/L)	Presence of *mcr-1*	Sequence type
**Reference strain**				
K-12 1655	Reference strain	0.25	–	ST10
J53	Containing pUHE-*gfp* recombinant plasmid	0.25	–	ST10
**CL-S**				
E015	Bacterial isolate from human blood	0.25	–	ST405
E139	Bacterial isolate from human blood	0.25	–	ST131
E154	Bacterial isolate from human blood	0.25	–	ST38
**CL-IR**				
E015R	Colistin-resistance-induced strain from E015	64	–	ST405
E139R	Colistin-resistance-induced strain from E139	>64	–	ST131
E154R	Colistin-resistance-induced strain from E154	64	–	ST38
**CL-chrR**				
QIA18	From Animal and Plant Quarantine Agency, Korea	8	–	ST226
QIA24	From Animal and Plant Quarantine Agency, Korea	8	–	ST4532
QIA32	From Animal and Plant Quarantine Agency, Korea	8	–	ST1
QIA33	From Animal and Plant Quarantine Agency, Korea	16	–	ST1
**CL-mcrR**				
EC006	Bacterial isolate from chicken	8	+	ST162
EC019	Bacterial isolate from chicken	8	+	ST410
EC111	Bacterial isolate from pig	16	+	ST1
**TC-mcr**				
J53_pEC__006_	Transconjugant containing pEC006	4	+	ST10
J53_pEC__019_	Transconjugant containing pEC019	4	+	ST10
J53_pEC__111_	Transconjugant containing pEC111	4	+	ST10

For genotyping of *E. coli* strains used in this study, sequence type (ST) were determined by multilocus sequence typing (MLST) analysis (Wirth et al., 2006). Amplicons were sequenced, and sequence types were predicted by using Enterobase.^1^

### *In vitro* Selection of Induced Colistin-Resistant Mutants and Bacterial Conjugation

CL-IR strains were obtained by serial passaging of CL-S strains with increasing concentration of colistin from 0.25to 64 mg/L. The colistin concentration was serially increased after every 24 h incubation. Spontaneous mutants were selected on Luria-Bertani (LB) agar plates containing 64 mg/L colistin (Lee et al., 2016).

To construct TC-*mcr* strains, conjugation was performed through the liquid mating method (Pham Thanh et al., 2016). CL-mcrR strains EC006, EC019, and EC111 were used as donors of *mcr-1*-harboring plasmids and *E. coli* J53 pUHE-gfp was employed as a recipient. Both donor and recipient were incubated in LB broth until optical density at 600 nm reached 0.5. These bacterial cultures were mixed using a 1:1 donor-to-recipient ratio and incubated overnight at 37°C. Transconjugants were selected on Mueller-Hinton (MH) agar plates with colistin (4 mg/L), sodium azide (100 mg/L), and 0.5 mM isopropyl β-D-1-thiogalacto-pyranoside (IPTG). The transconjugant colonies showed fluorescence in this selective media due to GFP (green fluorescent protein) induction by IPTG. To confirm the successful transfer of *mcr-1*-harboring plasmids, *mcr-1* was amplified and detected by primers CLR5_F and CLR5_R (Liu et al., 2016).

### Antimicrobial Susceptibility Testing

Minimum inhibitory concentration (MIC) was determined by the broth microdilution method according to Clinical and Laboratory Standards Institute (CLSI) guidelines (Clinical and Laboratory Standards Institute [CLSI], 2018). Twelve antibiotics including colistin, polymyxin B, imipenem, amikacin, cefepime, ceftriaxone, ciprofloxacin, tetracycline, rifampin, piperacillin/tazobactam, and ampicillin/sulbactam were tested. Susceptibility was defined according to CLSI breakpoints (Clinical and Laboratory Standards Institute [CLSI], 2018). *E. coli* ATCC 25922 and *Pseudomonas aeruginosa* ATCC 27853 were employed as reference strains.

### Amino Acid Alteration and mRNA Expression Analysis

PCR and DNA sequencing were performed to identify nucleotide (and resultant amino acid) substitutions in PmrAB, PhoPQ, and EptA in CL-S, CL-IR, CL-chrR, and CL-mcrR strains, using the primers listed in Supplementary Table S1. The gene expression levels of *pmrA*, *phoP*, and *eptA* were evaluated by quantitative real-time PCR (qRT-PCR; Supplementary Table S2). Total RNA was harvested from mid-log phase bacterial culture using RNeasy Mini Kit (Qiagen, Germany) and reverse transcription was performed using Omniscript Reverse Transcriptase (Qiagen, Germany). SYBR Green PCR Master Mix (Applied Biosystems, Foster City, CA, United States) and qRT-PCR primers were mixed with the complementary DNA to detect PCR amplification. qRT-PCR was conducted by using a Thermal Cycler Dice^TM^ Real Time System TP800 (Takara, Otsu, Japan). The expression level of *rrsB*, a housekeeping gene of *E. coli*, was evaluated to normalize the transcript levels. All tests were performed in triplicate.

### Plasmid Stability Assay

CL-mcrR strains were subjected to plasmid stability assays as described previously (Bryksin and Matsumura, 2010). Ten microliter overnight seed cultures of the strains were inoculated in 10 mL LB medium without antibiotic (1:1000 ratio). Subculturing was performed after 24 h incubation and repeated for 10 consecutive days. The total population of each culture was enumerated by plating onto a blood agar plate (BAP) without colistin. Identification of *mcr-1*-loss cells within each culture was determined by transferring 96 colonies from BAP to 4 mg/L colistin-containing MH broth in each well of a 96-well plate. The proportion of plasmid-containing cells were calculated by counting the number of wells that had visible growth of bacteria.

### Lipid A Structure Analysis

One CL-S strain (E015), one CR-IR strain (E015R), three CL-mcrR strains, one TC-mcr strain (J53_pEC__019_), and an *E. coli* reference strain J53 were subjected to lipid A structure analysis. Lipid A was extracted by ammonium hydroxide-isobutyric acid method (El Hamidi et al., 2005). Briefly, lyophilized cells (10 mg) were resuspended in 400 μL of isobutyric acid:1 M ammonium hydroxide (5:3 [vol/vol]) and incubated at 100°C for 4 h. The resuspensions were centrifuged for 15 min at 13,000 rpm, and supernatants were diluted in an equal volume of distilled water (DW) and lyophilized. Samples were washed with methanol and centrifuged for 15 min at 4,000 rpm. Insoluble lipid A was solubilized in a chloroform:methanol:DW mixture (3:1:0.25 [vol/vol/vol]). These lipid A suspensions were analyzed by negative-ion matrix-assisted laser desorption ionization time-of-flight (MALDI-TOF) mass spectrometry (Beceiro et al., 2011).

### Determination of Growth Curves and *in vitro* Competition Assay

Growth curves were determined by measuring the optical density at 600 nm using a spectrophotometer. Seed cultures of bacterial strains were inoculated in LB broth at a 1:100 ratio. The aliquots were collected at 0, 0.5, 1, 1.5, 2, 4, 6, 8, 12, 24, and 30 h.

All colistin-resistant and colistin-susceptible strains except TC-*mcr* strains were subjected to *in vitro* competition assay against *E. coli* K-12 MG1655. For TC-*mcr* strains, *E. coli* J53 was competed. Strains were cultured in LB broth until mid-log phase and mixed with MG1655 or J53 at a 1:1 ratio. The mixtures were incubated for 24 h at 37°C and shaking. After incubation, MH agar with and without colistin (4 mg/L) were used for plating dilutions of the bacterial mixture to differentiate the colistin-resistant strains and MG1655. In competition assay of colistin-susceptible (CL-S) strains against MG1655, ampicillin (100 mg/L) were used instead of colistin. Colonies were counted after overnight incubation and competition index (CI) values were determined based on (CFUs of colistin-resistant strain/CFUs of MG1655 or J53) ratio (Humphrey et al., 2012; Beceiro et al., 2014). Four independent competition experiments were performed to calculate the median CI value.

### Biofilm Formation and Serum Resistance Assay

Microtiter dish biofilm formation assays were employed to determine the capacity of biofilm formation, with minor modifications (Choi and Ko, 2015). In brief, strains were grown in LB broth with 0.5% glucose and the cultures were incubated until mid-log phase then inoculated into a 96-well flat-bottom plate. For quantitative assays, eight replicates were prepared and the inoculums were incubated for 24 h at 37°C. After incubation, cells were dumped out by turning the plate and washed once with water. A solution of crystal violet (0.1%) was added into each well and incubated at room temperature for 15 min. The plate was then rinsed two to three times with water and dried completely. Dried biofilm was solubilized by 95% ethanol and the absorbance at 600 nm was measured using a microplate reader (Bio-Rad, United States).

Bacterial susceptibility to the bactericidal activity of serum was measured by evaluating the surviving bacterial cells after incubation in diluted serum, as described previously (Siu et al., 2011). The bacterial cultures were incubated until mid-log phase in LB broth. One hundred μL of culture was washed once with 1X PBS and these bacterial suspensions were then mixed independently with 900 μL of 1/5 diluted normal human serum (NHS; Innovative Research, MI, United States) (NHS 180 μL + 1X PBS 720 μL) and incubated shaking for 3 h. After incubation, the mixture washed once with 1 × PBS and diluted for plating on BAP. Colonies were counted to determine survival rate in serum. Heat-inactivated NHS (30 min at 56°C) was employed as a positive control.

### Bacterial Survival in Macrophages

The macrophage cell line J774A.1, which is derived from BALB/c mice, was used for bacterial infection (Choi et al., 2014). Macrophages were maintained in Dulbecco’s Modified Eagle medium (DMEM; Welgene) supplemented with 10% heat-inactivated fetal bovine serum (FBS; Gibco) and 1% antibiotics-antimycotics (Thermo). The day before infection, 100 μL of J774A.1 cells (2.5 × 10^5^ cells) were plated with 400 μL of DMEM in a 24-well tissue culture plate, then incubated for 24 h for cell duplication and attachment. After incubation, the wells were washed three times with Dulbecco’s phosphate buffered saline (DPBS; Welgene) and 400 μL of DMEM only supplemented with 10% heat-inactivated FBS was added. Prior to bacterial infection, overnight-incubated bacterial cells were appropriately diluted to reach 5 × 10^7^ cells/mL. Diluted bacterial suspension (100 μL) was added for infection (multiplicity of infection: 20) after the cells were incubated for 1 h. After 30 min incubation for bacterial invasion of macrophages, the cells were washed with DPBS and then incubated in medium supplemented with 150 μg/mL gentamicin to remove extracellular bacteria. Following 1 h incubation, the cells were washed with DPBS and then incubated in medium containing 15 μg/mL of gentamicin. For the 0 h time point sample, the wells were washed and treated immediately by aspirating the medium and adding 500 μL of 1% Triton X-100 and 500 μL of DPBS. For the 4 h time point samples and onward, Triton X-100 was added at the desired time points. The content of each well was then diluted in DPBS and appropriate dilutions were plated on blood agar containing appropriate antibiotics. The percentage survival was obtained by dividing the number of bacteria recovered after 4 h, by the number of bacteria present at time 0. All media and DPBS were prewarmed in 37°C before use, and all the experiments were performed in duplicate.

### Bacterial Infection of *Drosophila melanogaster* (Fruit Fly)

Fly infection was performed by the thoracic needle pricking method (Heo et al., 2009). *D. melanogaster* Canton-S was used and 4- to 7-day-old female flies were only selected for fly infection. Fifteen flies were infected per strain. Bacterial strains were prepared by incubating until early stationary phase in LB broth. The cultures were washed once and resuspended on 1 × PBS. Flies were anesthetized with CO_2_ and placed on the CO_2_ pad. An insulin syringe with Ultra-Fine^TM^ needle (BD Bioscience) was used for pricking the thorax of the flies. The tip of the needle was dipped into the bacterial suspension and the very tip of the needle was inserted into the dorsolateral thorax. Infected flies were transferred into vials grouped by the infected strain. A pure PBS injection was used as a negative control and the fly mortality was monitored for up to 72 h post-infection. Death of the files was monitored and counted to determine the survival rate.

After 72 h incubation, four live flies per bacterial strain were selected to assess the bacterial proliferation *in vivo*. Flies were homogenized in 100 μL of 1 × PBS with a polypropylene pestle. Each homogenate was serially diluted and plated onto BAP agar containing colistin. The plates were incubated 37°C for 24 h and the number of CFUs per fly was counted. Each experiment was performed four times for all strains included in this study.

### Statistical Analyses

The data are presented as the mean ± SD. Statistical analyses were performed using Prism version 3.00 for Windows (GraphPad Software, San Diego, CA, United States). The differences were assessed using the Student’s *t*-test and one-way ANOVA. *P*-values < 0.05 were considered statistically significant.

## Results

### Characterization of *E. coli* Strains Used in This Study

Three CL-S strains, of which colistin MIC was 0.25 mg/L, belonged to different STs (ST405, ST131, and ST38) (Table 1). CL-IR strains induced from the CL-S strains showed high-level colistin resistance (MICs ≥ 64 mg/L). Four CL-chrR strains, which were colistin-resistant but *mcr*-negative, had an MIC of 8 to 16 mg/L of colistin and belonged to three different STs (ST226, ST4532, and ST1). CL-mcrR strains, *mcr-1*-positive strains isolated from livestock, also had an MIC of 8 to 16 mg/L of colistin and belonged to different STs (ST162, ST410, and ST1). Colistin MICs of TC-mcr strains, transconjugants with *mcr-1*-bearing plasmids, were 4 mg/L.

All strains were susceptible to imipenem, but resistant to ampicillin/sulbactam (Supplementary Table S3). All CL-S strains were resistant to cefepime and ceftriaxone. Although E015R was resistant to cefepime and ceftriaxone (as its parent strain, E015), the other CL-IR strains (E0139R and E154R) were susceptible to them. In addition, E154R was susceptible to ciprofloxacin despite the ciprofloxacin resistance of E154. While CL-chrR strains were susceptible to cefepime, ceftriaxone (except QIA18), and ciprofloxacin, CL-mcrR strains were resistant to them. However, TC-mcr strains with *mcr-1*-positive plasmids of CL-mcrR strains were susceptible to them. Meanwhile, CL-mcrR strains, except EC006, were susceptible to tetracycline, but CL-chrR strains were resistant.

### Amino Acid Alteration of PmrAB, PhoPQ, and EptA

Amino acid sequences of PmrAB and PhoPQ, which are well-known two-component regulatory systems associated with colistin resistance, were constructed from determined nucleotide sequences (Table 2). CL-IR strains had amino acid alterations in PmrB, compared with their parental CL-S strains: VSRL_133__–__136_ deletion in E015R, P94L substitution in E139R, and A159V substitution in E154R. All but one CL-chrR strains showed amino acid alterations in PmrB and PhoQ not found in CL-S strains: D101E and V386L in PhoQ of QIA18, H2R, L14Q, and S138N in PmrB and S138T in PhoQ of QIA32 and QIA33. No amino acid alterations were identified in PmrAB and PhoPQ of QIA24. Amino acid substitutions found in CL-chrR strains, QIA32 and QIA33, such as H2R and S138N in PmrB and S138T in PhoQ, were also identified in one CL-mcrR strain, EC111.

**TABLE 2:** Amino acid alterations of PmrAB and PhoPQ.^a^

*E. coli* strain	PmrA		PmrB											PhoP	PhoQ		
				
	31	144	2	14	94	108	123	133–136	138	159	283	351	358	44	101	138	386
				
K-12 MG1655	T	G	H	L	P	D	E	VSRL	S	A	D	V	Y	I	D	S	V
**CL-S**																	
E015		S									G			L			
E139	S						D					I		L			
E154											G			L			
**CL-IR**																	
E015R		S						**DEL**^*b*^			G			L			
E139R	S				**L**		D					I		L			
E154R										**V**	G			L			
**CL-chrR**																	
QIA18															**E**		**L**
QIA24											G			L			
QIA32			**R**	**Q**					**N**		G			L		**T**	
QIA33			**R**	**Q**					**N**		G			L		**T**	
**CL-mcrR**																	
EC006													N				
EC019		S				N							N				
EC111			**R**			N			**N**							**T**	

*^*a*^Amino acid alterations found only in colistin-resistant strains are indicated in bold. ^*b*^DEL, deletion.*

Amino acid alterations in EptA, a homolog of PmrC, were also identified in CL-S, CL-IR, CL-chrR, and CL-mcrR strains. A CL-IR strain, E139R, had many amino acid differences in EptA from its colistin-susceptible, parental strain (Table 3). In addition, two amino acid alterations in EptA, T148A or K233T, were identified in a CL-IR strain (E154R), two CL-chrR strains (QIA32 and QIA33), and a CL-mcrR strain (EC111).

**TABLE 3:** Amino acid alterations of EptA.^a^

*E. coli* strain	EptA																							
	
	14	15	21	26	39	69	106	123	130	137	147	148	163	211	217	232	233	332	341	348	366	411	413	414
	
K-12 MG1655	L	A	A	I	A	S	A	Q	F	L	A	T	V	L	V	E	K	A	V	D	E	T	T	K
**CL-S**																								
E015											T									G				
E139																				G			S	
E154																				G				
**CL-IR**																								
E015R											T									G				
E139R	**F**	**S**	**T**	**V**	**V**	**G**	**T**	**R**	**L**	**I**	T		**I**	**S**	**I**	**G**		**V**		G	**D**			**Q**
E154R																	**T**			G				
**CL-chrR**																								
QIA18																				G			S	
QIA24																				G			S	
QIA32												**A**					**T**			G				
QIA33												**A**					**T**			G				
**CL-mcrR**																								
EC006																			I	G		I	S	
EC019																				G			S	
EC111												**A**					**T**			G				

*^*a*^Amino acid alterations found only in colistin-resistant strains are indicated in bold.*

Two substitutions in PmrB of CL-mcrR strains, D108N and Y358N, were found only in the *mcr-1*-positive strains, thus they could not be considered to contribute colistin resistance in the strains (Table 2). The same was for the two substitutions in EptA, V341I, and T411I (Table 3).

### mRNA Expression of pmrA, phoP, and eptA

**FIGURE 1:**
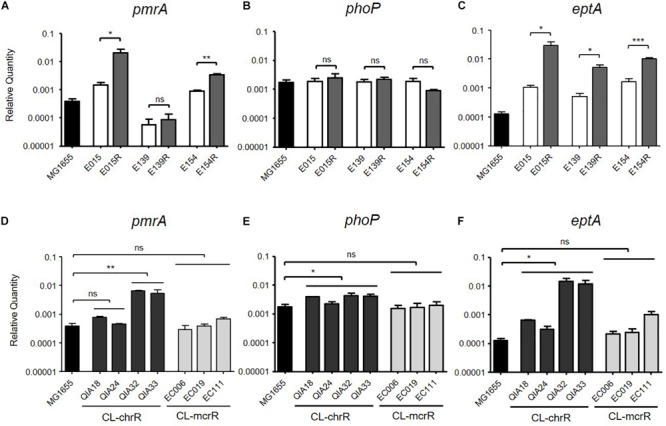
mRNA expression levels of *pmrA, phoP*, and *eptA*. The mRNA expression was measured by qRT-PCR. **(A–C)** Comparison of expression levels of *pmrA, phoP*, and *eptA* between CL-S and CL-IR strains. The expression levels in a reference strain MG1655 are also presented. **(D–F)** Comparison of expression levels of *pmrA, phoP*, and *eptA* between a reference strain MG1655, CL-chrR, and CL-mcrR strains. Statistics were done with unpaired, two-tailed *t*-test. *P ≤ 0.05; **P ≤ 0.01; ***P ≤ 0.0001; ns, not significant.

Two CL-IR strains, E015R and E154R, showed elevated mRNA expression of *pmrA*, compared to their parental CL-S strains (Figure 1A), but a remaining CL-IR strain, E139R, showed no difference in mRNA expression of *pmrA* with E139. However, mRNA expression of *eptA* in E139R increased as in the other CL-IR strains (Figure 1C). No difference in mRNA expression level was not found in *phoP* between CL-S and CL-IR strains (Figure 1B).

mRNA expression levels of *pmrA, phoP*, and *eptA* were compared between CL-chrR and CL-mcrR strains (Figures 1D–F). While CL-chrR strains showed elevated mRNA expression of *phoP* and *eptA* compared with a reference strain MG1655, no difference in mRNA expression of *pmrA, phoP*, and *eptA* was shown in CL-mcrR strains. The mRNA expression levels of *pmrA* in CL-chrR strains were different according to strains (Figure 1D).

### Plasmid Stability

**FIGURE 2:**
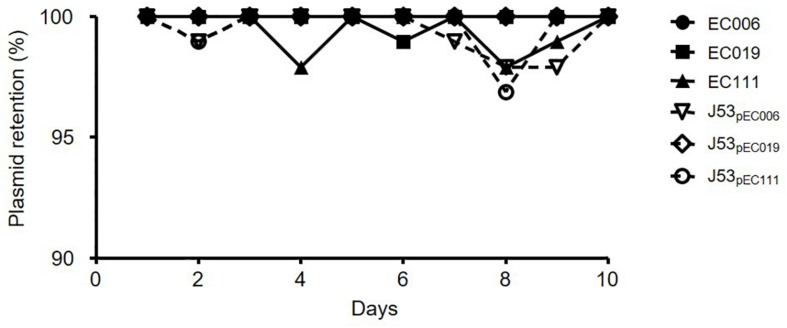
Plasmid stability of *mcr-1*-harboring *E. coli* strains. Plasmid stability was determined by colony picking onto selective media in 96-well plates and is presented as a percentage of the wells with bacterial growth on media with colistin.

As a result of plasmid stability assays for CL-mcrR and TC-mcr strains, most populations of six strains cultured in antibiotic-free media could grow on media with 4 mg/L colistin for 10 consecutive days (Figure 2). The live colonies in media with colistin contained *mcr-1*-bearing plasmid by PCR.

### Growth Rates and *in vitro* Competition Assay

**FIGURE 3:**
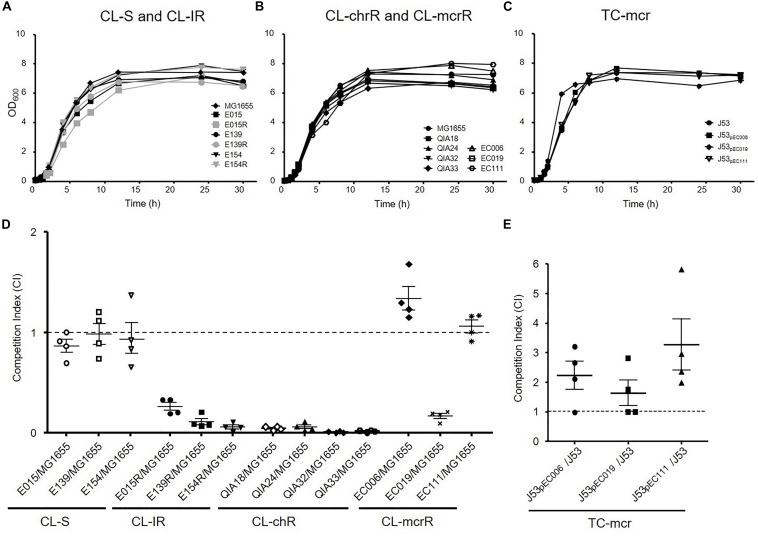
Growth rates and fitness cost. **(A–C)** Growth curves of CL-S, CL-IR, CL-chrR, CL-mcrR, and TC-mcr strains. *y* axis, optical densities (OD) of broth cultures at 600 nm; *x* axis, time of growth (hours). **(D)** *In vitro* competitive fitness of CL-S, CL-IR, CL-chrR, and CL-mcrR strains, which were competed with a reference *E. coli* strain MG1655. While CL-S strains were differentiated from MG1655 in MH agar with or without ampicillin (100 mg/L), the others were in agar with or without colistin (4 mg/L). **(E)** Competitive fitness of three transconjugants (TC-mcr strains), which competed with their recipient strain, J53. A CI value less than 1 indicates a fitness defect, and a value greater than 1 indicates a fitness benefit. The error bars indicate the standard deviations.

To investigate whether colistin resistance affects fitness burden compared to colistin susceptible strains, growth curves and *in vitro* competition assays were performed (Figure 3). The growth rates of CL-IR strains were not different compared to their colistin-susceptible parent strains (Figure 3A). No significant difference in growth rates was found between CL-chrR and CL-mcrR strains (Figure 3B). In addition, the TC-mcr strains with additional plasmid bearing *mcr-1* showed similar growth rates to recipient strain J53 (Figure 3C).

In the antibiotic-free media, CL-S, CL-IR, CL-chrR, and CL-mcrR strains competed with the colistin-susceptible reference *E. coli* strains MG1655 derived from K-12 or J53, respectively (Figure 3D). While CL-S strains showed similar competitiveness against MG1655, all CL-IR and CL-chrR strains showed CI values of less than 1, that is, they were less competitive than the susceptible strain. However, CL-mcrR strains did not: while EC019 had a competitive disadvantage, the other two CL-mcrR strains (EC006 and EC111) outcompeted the colistin-susceptible strain MG1655, showing CI values > 1. The TC-mcr strains competed with their parental plasmid-free *E. coli* strain J53 (Figure 3E). As a result, all three TC-mcr strains were significantly competitive than the J53 strain.

### Biofilm Formation and Serum Resistance

**FIGURE 4:**
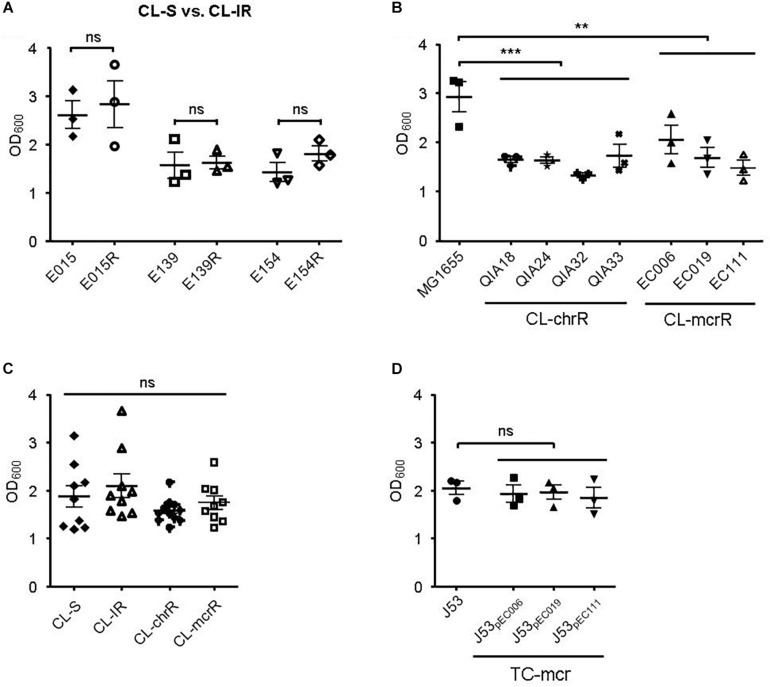
Results of biofilm formation. **(A)** Comparison of biofilm formation between CL-S and CL-IR strains. **(B)** Biofilm formation of CL-chrR and CL-mcrR strains was compared with that of *E. coli* reference strain MG1655. **(C)** Comparison of biofilm formation among CL-S, CL-IR, CL-chrR, and CL-mcrR strains. **(D)** Biofilm formation of TC-mcr strains was compared with that of their recipient strain, J53. The error bars indicate the standard deviations. Statistical analyses were conducted with unpaired, two-tailed *t*-test. **P ≤ 0.01; ***P ≤ 0.0001; ns, not significant.

First, we compared biofilm formation between CL-S and CL-IR strains (Figure 4A). The CL-IR strains showed no difference of biofilm formation compared with their parental strains. Both CL-chrR strains and CL-mcrR strains formed considerably less biofilm than the reference strain MG1655, but no difference in biofilm formation was measured between them (Figure 4B). Four groups of CL-S, CL-IR, CL-chrR, and CL-mcrR had similar biofilm formation (Figure 4C). In addition, the TC-mcr strains formed similar biofilms to their recipient strain J53 (Figure 4D).

**FIGURE 5:**
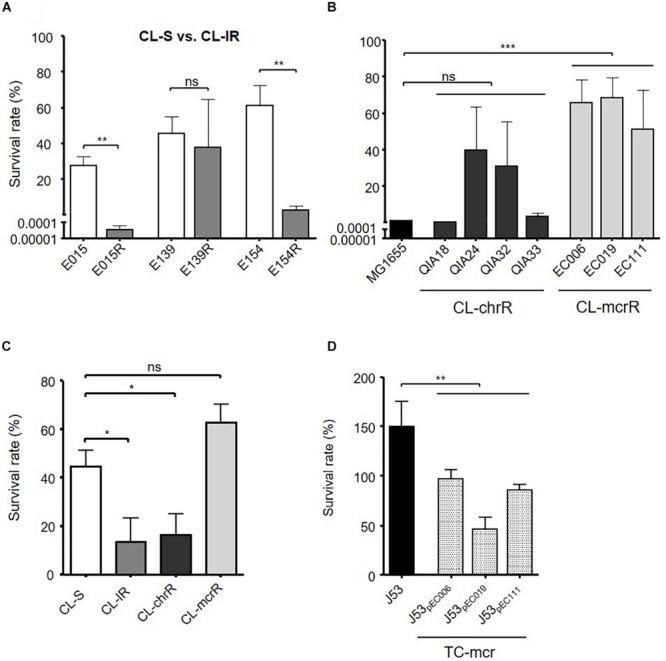
Results of serum resistance assay. **(A)** Comparison of survival rates in human serum between CL-S and CL-IR strains. **(B)** Survival rates of CL-chrR and CL-mcrR strains in human serum were compared with that of *E. coli* reference strain MG1655. **(C)** Comparison of survival rates in human serum among CL-S, CL-IR, CL-chrR, and CL-mcrR strains. **(D)** Survival rates of TC-mcr strains in human serum were compared with that of their recipient strain, J53. The error bars indicate the standard deviations. Statistics were conducted with unpaired, two-tailed *t*-tests. *P ≤ 0.05; **P ≤ 0.01; ***P ≤ 0.0001; ns, not significant.

Contrary to the similar biofilm formation among bacterial groups, serum resistance varied between the groups (Figure 5). Two CL-IR strains (E015R and E154R) had a decrease in survival rates in human serum compared with their parental CL-S strains (Figure 5A). The CL-mcrR strains had very high survival rates in human serum, although CL-chrR strains had no significant difference in serum resistance compared to MG1655 (Figure 5B). That is, CL-IR and CL-chrR strains showed less resistance to human serum than CL-S strains, but CL-mcrR strains showed similar serum susceptibility to CL-S strains (Figure 5C). Although TC-mcr strains did not show significantly different survival rates in human serum from CL-mcrR strains (62.97 ± 20.65% vs. 76.21 ± 26.75%, *P*-value 0.2758), they were much less than that of plasmid-free parental strain J53 (Figure 5D).

### Bacterial Survival in Macrophages

**FIGURE 6:**
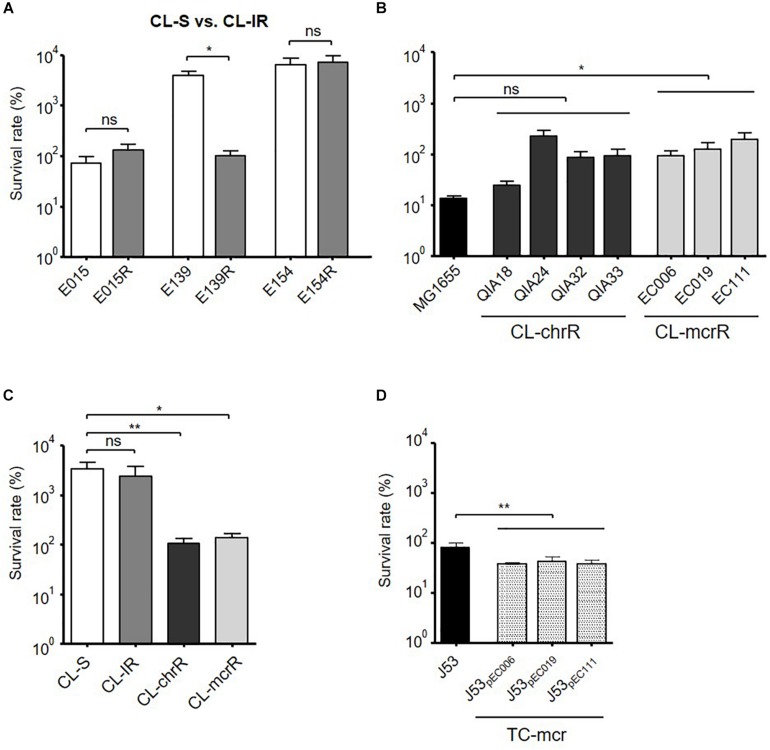
Bacterial survival rates in macrophages. Survival rates of bacterial strains inside macrophages (J774A.1), which were measured at 4 h of infection. **(A)** Comparison of survival rates in macrophages between CL-S and CL-IR strains. **(B)** Survival rates of CL-chrR and CL-mcrR strains in macrophages were compared with that of *E. coli* reference strain MG1655. **(C)** Comparison of survival rates in macrophages among CL-S, CL-IR, CL-chrR, and CL-mcrR strains. **(D)** Survival rates of TC-mcr strains in macrophages were compared with that of their recipient strain, J53. The error bars indicate the standard deviations. Statistics were conducted with unpaired, two-tailed *t*-test. *P ≤ 0.05; **P ≤ 0.01; ns, not significant.

Bacterial survival inside macrophages was compared (Figure 6). While E139R had significantly reduced survival rate in macrophages compared with its isogenic susceptible strain, E139, the survival rates of the other CL-IR strains were not different from those of CL-S strains (Figure 6A). CL-chrR strains did not have different survival rates in macrophages from MG1655, but CL-mcrR strains survived better than MG1655 (Figure 6B). However, both CL-chrR and CL-mcrR strains exhibited lower survival rates than CL-S and CL-IR strains (Figure 6C). The survival rates of transconjugant strains, TC-mcr, were significantly low compared with that of their plasmid-free parental strain J53 (Figure 6D).

### Fruit Fly Infection

**FIGURE 7:**
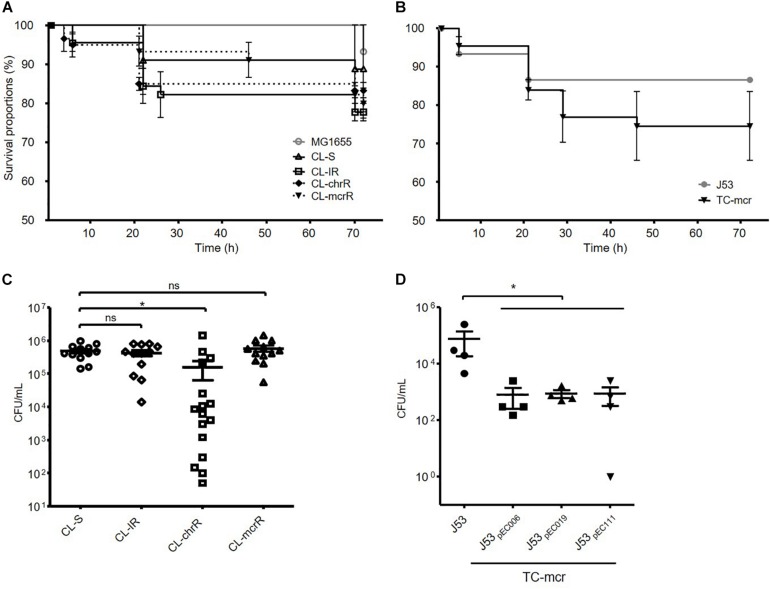
Results of fruit fly (*D. melanogaster*) infections. **(A,B)** Survival rates of flies infected with bacterial isolates obtained after incubation for 15 h. 15 flies were infected with each strain. **(C,D)** Number of surviving colonies of bacteria in the flies after 72 h of infection. Four fruit flies were used for each strain, and dots indicate CFUs in a single fly. The error bars indicate the standard deviations. Statistics were conducted with unpaired, two-tailed *t*-tests. *P ≤ 0.05; ns, not significant.

We examined the survival rates of *D. melanogaster* infected with *E. coli*. Three groups of colistin-resistant strains, CL-IR, CL-chrR, and CL-mcrR strains, did not show different fly killing ability compared to the CL-S strains and a reference strain MG1655 (Figure 7A). The transconjugants with a *mcr-1*-bearing plasmid decreased survival rates of *D. melanogaster* than their parental strain J53, which was not significant (*P*-value 0.27) (Figure 7B).

In addition, we measured the number of viable bacterial populations from flies after 72 h of infection. Significantly less bacterial colonies survived in the flies infected with CL-chrR strains than CL-S strains, but no difference was measured among CL-S, CL-IR, and CL-mcrR strains (Figure 7C). However, the transconjugants, TC-mcr strains, had a lower bacterial load in infected *D. melanogaster* than their parental strain J53 (Figure 7D).

## Discussion

In this study, we compared fitness cost and virulence between colistin-resistant *E. coli* strains due to chromosomal gene mutations and plasmid-borne *mcr-1* gene. In addition to wild-type colistin-resistant strains (CL-chrR and CL-mcrR), *in vitro*-selected colistin-resistant strains (CL-IR) and transconjugants with *mcr-1*-carrying plasmid (TC-mcr) were included.

First, we identified several mutations in two-component regulatory systems, PmrAB and PhoPQ, in colistin-resistant strains included in this study. However, not all mutations are associated with colistin resistance. H2R in PmrB and V386L in in PhoQ, which were identified in only colistin-resistant strains in this study, were reported to not be associated with colistin resistance in *E. coli* in previous studies (Luo et al., 2017; Sato et al., 2018). Although E123D in PmrB was reported to be associated with colistin resistance in a previous study (Luo et al., 2017), it was found in a colistin-susceptible strain in this study. Y358N in PmrB was identified in CL-mcrR strains in this study, but it was reported to contribute to colistin resistance in the previous study (Luo et al., 2017). While S138T in PhoQ was mentioned to be associated with colistin resistance in one study (Delannoy et al., 2017), it was not in another study (Luo et al., 2017). Amino acid substitutions in L14, P94, and A159 of PmrB were also identified recently in colistin-resistant *E. coli* isolates from France, but their association with colistin resistance were not confirmed (Bourrel et al., 2019). Thus, amino acid alterations contributing colistin resistance in *E. coli* are not well-established, and further studies are required.

Gene expression comparison showed that colistin resistance in CL-IR and CL-chrR strains may be ultimately due to overexpression of *eptA*. For CL-IR strains, overexpression of *pmrA* was shown in two strains, but no strains showed overexpression of *phoP*. However, overexpression of *eptA* was identified in all three CL-IR strains. In addition, *eptA* was overexpressed significantly in CL-chrR strains, although *pmrA* overexpression was not observed in two CL-chrR strains. In particular, two amino acid alterations in *eptA* (T148A and K233T) are probably associated with overexpression on the gene, which results in colistin resistance, because the strains with the mutations (E154R, QIA32, QIA33, and EC111) showed obvious high expression of *eptA*. The association of *eptA* mutation and colistin resistance has been suggested in several Gram-negative bacteria, including *E. coli*, *K. pneumoniae*, and *A. baumannii* (Lesho et al., 2013; Mathur et al., 2018; Xu et al., 2018; Gerson et al., 2019). The high variation of *eptA* in E139R may be due to genetic exchange of chromosomal portions including *eptA*, but its donor could not be identified. The overexpression of *eptA* in the strain EC111 may explain its high colistin MIC relative to the other *mcr-1*-positive strains.

Fitness and virulence of colistin resistance due to chromosomal mutations has previously been explored mainly in *A. baumannii* and *K. pneumoniae*. Some studies have reported defective growth rates and reduced virulence in colistin-resistant *A. baumannii* (López-Rojas et al., 2011, 2013; Pournaras et al., 2014). Others have reported colistin resistance without loss of fitness and virulence (Cannatelli et al., 2015; Durante-Mangoni et al., 2015; Wand et al., 2015). However, several papers have revealed that fitness and virulence in colistin-resistant *A. baumannii* may be affected by resistance mechanisms; while colistin resistance due to mutations in *pmrAB* showed no loss of fitness and virulence, the strains with impaired LPS due to mutations in *lpx* genes suffered from a fitness cost and reduced virulence (Beceiro et al., 2014; Mu et al., 2016).

The impact of colistin resistance due to chromosomal mutations on bacterial fitness and virulence has been rarely investigated in *E. coli*, but several studies have been reported on the fitness and virulence of *mcr-1*-mediated colistin resistance (Zhang et al., 2017; Ma et al., 2018; Tietgen et al., 2018; Wang et al., 2018; Wu et al., 2018). Several studies showed no considerable fitness cost due to acquisition of *mcr-1*-bearing plasmids in *E. coli* (Zhang et al., 2017; Tietgen et al., 2018; Wang et al., 2018) and Ma et al. (2018) reported that an *mcr-1*-bearing plasmid initially caused a fitness cost, but such cost was largely reduced after serial cultures. It was also reported that fitness would be different according to plasmids bearing *mcr-1* (Wu et al., 2018). In addition, it was revealed that *mcr-1* imposed fitness cost in *K. pneumoniae* (Nang et al., 2018; Tietgen et al., 2018).

Our results showed that colistin-resistant *E. coli* strains with chromosomal mutations as well as wild-type colistin-resistant and resistance-induced strains have a fitness cost based on *in vitro* competition assays, although they did not show defect in growth rate. On the other hand, all *mcr-1*-positive *E. coli* strains, except one strain from chicken (EC019), did not show fitness cost. It is known that mutations and altered expression in PhoPQ or PmrAB and EptA facilitate the addition of PEtN to lipid A of LPS, resulting in colistin resistance in Gram-negative bacteria (Jeannot et al., 2017). In the colistin-resistant *E. coli* strains included in this study, we identified the addition of PEtN by MALDI-TOF mass spectrometry (Supplementary Figure S1). It is assumed that the additional PEtN would be a fitness burden in *E. coli*, explaining the low fitness of colistin-resistant strains due to chromosomal mutations. The fitness cost in colistin-resistant strains due to chromosomal mutations may explain why colistin resistance in Gram-negative bacteria has not increased sharply.

On the other hand, colistin-resistant *E. coli* strains with *mcr-1*-harboring plasmids did not suffer a fitness cost, which might be consistent with rapid dissemination of *mcr*-positive strains (Sun et al., 2018). Certain genes in the plasmid with *mcr-1* may compensate for the fitness cost in colistin-resistant strains. Thus, the effect of a plasmid on bacterial host’s fitness may be different according to type of plasmid and bacterial host, which is consistent with the results in this study as well as in the previous studies (Zhang et al., 2017; Ma et al., 2018; Tietgen et al., 2018; Wang et al., 2018; Wu et al., 2018). Which components of the plasmid confer the compensation against fitness cost to bacterial hosts should be further studied.

No difference in biofilm formation was found among the groups, which was expected because the addition of PEtN to lipid A in cell membranes does not affect biofilm formation and no genes associated with biofilm formation were found in the plasmids (Lee et al., 2018). However, virulence measured by serum resistance and bacterial survival in macrophages and fruit fly was different among the groups. The results revealed that colistin resistance in *E. coli* brings about loss of virulence without respect to cause of resistance. Both CL-IR and CL-chrR strains showed lower survival rates in human serum than CL-S strains. Although CL-IR strains showed similar survival rates in macrophages and flies to CL-S strains, the survival of CL-chrR strains was significantly defective. The *mcr-1*-positive, colistin-resistant strains from livestock (CL-mcrR strains) showed similar survival rates to CL-S strains in human serum and in flies, but the survival rates of transconjugants with *mcr-1*-harboring plasmids in human serum and in macrophages decreased greatly compared with their parental strains. In macrophages, both CL-mcrR and TC-mcr strains had significantly reduced survival rates. Taken together, both chromosomal mutations causing colistin resistance and the introduction of *mcr-1*-bearing plasmid reduces virulence in *E. coli*.

Although our study showed overall reduced virulence in colistin-resistant strains, the change of virulence may be strain-dependent rather than associated with resistance mechanisms (Wand et al., 2015). For example, an *mcr-1*-positive strain, EC019, showed less *in vitro* competitiveness compared with the other *mcr-1*-positive strains. In addition, diverse survival rates in human serum, macrophages, and fruit flies were identified among the CL-IR and CL-chrR strains. Because the *E. coli* isolates included in this study belonged to diverse clones with different STs, it may not be excluded the possibility that the fitness cost and virulence were associated with their genotypes, which would be investigated further.

It is not clear what affects the virulence in colistin-resistant strains. While the change of the cell membrane due to the addition of PEtN confers bacterial resistance to innate immune defensins and colistin, it may affect the survival of bacteria in other hostile environments. In addition, it has been reported that colistin resistance may affect the amount of polysaccharide in the cell wall of *K. pneumoniae*, which may affect the its virulence (Choi and Ko, 2015).

In conclusion, our study demonstrates that colistin-resistant *E. coli* strains due to chromosomal mutations have a fitness cost, but plasmids bearing *mcr-1* do not give rise to a fitness burden in *E. coli*, despite an exception. In addition, we show that virulence measured by resistance to human serum and survival in the macrophage and fruit fly was lowered both in colistin-resistant *E. coli* strains due to chromosomal mutations and *mcr-1*-harboring plasmids. Along with high usage of polymyxins in both agriculture and healthcare sectors, the lack of fitness cost of *mcr-1*-positive strains may facilitate rapid spread of colistin resistance.

## Data Availability Statement

The raw data supporting the conclusions of this article will be made available by the authors, without undue reservation, to any qualified researcher.

## Ethics Statement

This study uses strains obtained from blood, obtained from ABB in APFID (Asian-Pacific Research Foundation of Infectious Disease, Seoul, South Korea). Sungkyunkwan University School of Medicine did not require the study to be reviewed or approved by an ethics committee because the strains were not collected for this study.

## Author Contributions

YC performed the experiments, analyzed the data, and wrote the manuscript. J-YL and HL performed the experiments and analyzed the data. MP, KJK, S-KL, and DS provided the experimental materials, analyzed the data. KSK designed the experiments, analyzed the data, and wrote the manuscript.

## Conflict of Interest

The authors declare that the research was conducted in the absence of any commercial or financial relationships that could be construed as a potential conflict of interest.

## Footnotes

1 http://enterobase.warwick.ac.uk

## References

[B1] Andersson D. I. (2006). The biological cost of mutational antibiotic resistance: any practical conclusions? *Curr. Opin. Microbiol.* 9 461–465. 16890008 10.1016/j.mib.2006.07.002

[B2] Beceiro A. Llobet E. Aranda J. Bengoechea J. A. Doumith M. Hornsey M. (2011). Phosphoethanolamine modification of lipid A in colistin-resistant variants of *Acinetobacter baumannii* mediated by the *pmrAB* two-component regulatory system. *Antimicrob. Agents Chemother.* 55 3370–3379. 21576434 10.1128/AAC.00079-11 PMC3122444

[B3] Beceiro A. Moreno A. Fernández N. Vallejo J. A. Aranda J. Adler B. (2014). Biological cost of different mechanisms of colistin resistance and their impact on virulence in *Acinetobacter baumannii*. *Antimicrob. Agents Chemother.* 58 518–526. 10.1128/AAC.01597-13 24189257 PMC3910726

[B4] Bourrel A. S. Poirel L. Royer G. Darty M. Vuillemin X. Kieffer N. (2019). Colistin resistance in Parisian inpatient faecal *Escherichia coli* as the result of two distinct evolutionary pathways. *J. Antimicrob. Chemother.* 74 1521–1530. 10.1093/jac/dkz090 30863849

[B5] Bryksin A. V. Matsumura I. (2010). Rational design of a plasmid origin that replicates efficiently in both gram-positive and gram-negative bacteria. *PLoS One* 5:e13244. 10.1371/journal.pone.0192508 20949038 PMC2951906

[B6] Cannatelli A. Santos-Lopex A. Glani T. Gonzalez-Zorn B. Rossolini G. M. (2015). Polymyxin resistance caused by *mgrB* inactivation is not associated with significant biological cost in *Klebsiella pneumoniae*. *Antimicrob Agents Chemother* 59 2898–2900. 25691629 10.1128/AAC.04998-14 PMC4394794

[B7] Choi E. Kim H. Lee H. Nam D. Choi J. Shin D. (2014). The iron-sensing Fur regulator controls expression timing and levels of *Salmonella* pathogenicity island 2 genes in the course of environmental acidification. *Infect. Immun.* 82 2203–2210. 10.1128/IAI.01625-13 24643535 PMC4019186

[B8] Choi M. J. Ko K. S. (2015). Loss of hypermucoviscosity and increased fitness cost in colistin-resistant *Klebsiella pneumoniae* ST23 strains. *Antimicrob. Agents Chemother.* 59 6763–6773. 10.1128/AAC.00952-15 26282408 PMC4604379

[B9] Clinical and Laboratory Standards Institute [CLSI] (2018). *Performance Standards for Antimicobial Susceptibility Testing: 26th Informational Supplement.* Wayne, PA: CLSI.

[B10] Delannoy S. Le Devendec L. Jouy E. Fach P. Drider D. Kempf I. (2017). Characerization of colistin-resistant *Escherichia coli* isolated from diseased pigs in France. *Front. Microbiol.* 8:2278 10.3389/fmicb.2017.02278 PMC5702452 29209292

[B11] Durante-Mangoni E. Del Franco M. Andini R. Bernardo M. Giannouli M. Zarrilli R. (2015). Emergence of colistin resistance without loss of fitness and virulence after prolonged colistin administration in a patient with extensively drug-resistant *Acinetobacter baumannii*. *Diagn. Microbiol. Infect. Dis.* 82 222–226. 10.1016/j.diagmicrobio.2015.03.013 25858028

[B12] El Hamidi A. Tirsoaga A. Novikov A. Hussein A. Caroff M. (2005). Microextraction of bacterial lipid a: easy and rapid method for mass spectrometric characterization. *J. Lipid Res.* 46 1773–1778. 10.1194/jlr.D500014-JLR200 15930524

[B13] Gerson S. Betts J. W. Lucaßen K. Nodari C. S. Wille J. Josten M. (2019). Investigation of novel *pmrB* and *eptA* mutations in isogenic *Acinetobacter baumannii* isolates associated with colistin resistance and increased virulence *in vivo*. *Antimicrob. Agents Chemother.* 63:e1586-18. 10.1128/AAC.01586-18 30617096 PMC6395940

[B14] Heo Y. J. Lee Y. R. Jung H. H. Lee J. Ko G. Cho Y. H. (2009). Antibacterial efficacy of phages against *Pseudomonas aeruginosa* infections in mice and *Drosophila melanogaster*. *Antimicrob. Agents Chemother.* 53 2469–2474. 10.1128/AAC.01646-08 19364866 PMC2687186

[B15] Hinchliffe P. Yang Q. E. Portal E. Young T. Li H. Tooke C. L. (2018). Insights into the mechanistic basis of plasmid-mediated colistin resistance from crystal structures of the catalytic domain of MCR-1. *Sci. Rep.* 7:39392. 10.1038/srep39392 28059088 PMC5216409

[B16] Humphrey B. Thomson N. R. Thomas C. M. Brooks K. Sanders M. Delsol A. A. (2012). Fitness of *Escherichia coli* strains carrying expressed and partially silent IncN and IncP1 plasmids. *BMC Microbiol.* 12:53. 10.1186/1471-2180-12-53 22475035 PMC3347995

[B17] Jeannot K. Bolard A. Plésiat P. (2017). Resistance to polymyxins in Gram-negative organisms. *Int. J. Antimicrob. Agents* 49 526–535. 28163137 10.1016/j.ijantimicag.2016.11.029

[B18] Landman D. Georgescu C. Martin D. A. Quale J. (2008). Polymyxins revisited. *Clin. Microbiol. Rev.* 21 449–465. 18625681 10.1128/CMR.00006-08 PMC2493081

[B19] Lee J. Y. Lim S. K. Choi Y. Moon D. C. Shin J. Ko K. S. (2018). Whole sequences and characteristics of *mcr-1*-harboring plasmids of *Escherichia coli* strains isolated from livestock in South Korea. *Microb. Drug Resist.* 24 489–492. 10.1089/mdr.2017.0369 29485936

[B20] Lee J. Y. Park Y. K. Chung E. S. Na I. Y. Ko K. S. (2016). Evolved resistance to colistin and its loss due to genetic reversion in *Pseudomonas aeruginosa*. *Sci. Rep.* 6:25543. 10.1038/srep30365 27150578 PMC4858706

[B21] Lesho E. Yoon E. J. McGann P. Snesrud E. Kwak Y. Milillo M. (2013). Emergence of colistin-resistance in extremely drug-resistant *Acinetobacter baumannii* containing a novel *pmrCAB* operon during colistin therapy of wound infections. *J. Infect. Dis.* 208 1142–1151. 23812239 10.1093/infdis/jit293

[B22] Liu Y. Y. Wang Y. Walsh T. R. Yi L. X. Zhang R. Spencer J. (2016). Emergence of plasmid-mediated colistin resistance mechanism MCR-1 in animals and human beings in China: a microbiological and molecular biological study. *Lancet Infect. Dis.* 16 161–168. 26603172 10.1016/S1473-3099(15)00424-7

[B23] López-Rojas R. Domínguez-Herrera J. McConnell M. J. Docobo-Peréz F. Smani Y. Fernández-Reyes M. (2011). Impaired virulence and in vivo fitness of colistin-resistant *Acinetobacter baumannii*. *J. Infect. Dis.* 203 545–548. 10.1093/infdis/jiq086 21216865 PMC3071218

[B24] López-Rojas R. McConnell M. J. Jiménez-Mejía M. E. Domínguez-Herrera J. Fernández-Reyes M. Rivas L. (2013). Colistin resistance in a clinical *Acinetobacter baumannii* strain appearing after colistin treatment: effect on virulence and bacterial fitness. *Antimicrob. Agents Chemother.* 57 4587–4589. 23836165 10.1128/AAC.00543-13 PMC3754302

[B25] Luo Q. Yu W. Zhou K. Guo L. Shen P. Lu H. (2017). Molecular epidemiology and colistin resistant mechanism of *mcr*-positive and *mcr*-negative clinical isolated *Escherichia coli*. *Front. Microbiol.* 8:2262 10.3389/fmicb.2017.02262 PMC5715374 29250039

[B26] Ma K. Feng Y. Zong Z. (2018). Fitness cost of a mcr-1-carrrying IncHI2 plasmid. *PLoS One* 13:e0209706 10.1371/journal.pone.01209706 PMC6306219 30586457

[B27] Mathur P. Veeraraghavan B. Devanga Ragupathi N. K. Inbanathan F. Y. Khurana S. (2018). Multiple mutations in lipid-A modification pathway & novel *fosA* variants in colistin-resistant *Klebsiella pneumoniae*. *Future Sci. OA* 4:FSO319. 10.4155/fsoa-2018-0011 30112189 PMC6088269

[B28] Mu X. Wang N. Li X. Shi K. Zhou Z. Yu Y. (2016). The effect of colistin resistance-associated mutations on the fitness of *Acinetobacter baumannii*. *Front. Microbiol.* 7:1715 10.3389/fmicb.2017.01715 PMC5088200 27847502

[B29] Nang S. C. Morris F. C. McDonald M. J. Han M. L. Wang J. Strugnell R. A. (2018). Fitness cost of mcr-1-mediated polymyxin resistance in *Klebsiella pneumoniae*. *J. Antmicrob. Chemother.* 73 1604–1610. 10.1093/jac/dky061 29514208 PMC6693033

[B30] Pham Thanh D. Thanh Tuyen H. Nguyen T. Nguyen T. Chung The H. Wick R. R. (2016). Inducible colistin resistance via a disrupted plasmid-borne *mcr-1* gene in a 2008 Vietnamese *Shigella sonnei* isolate. *J. Antimicrob. Chemother.* 71 2314–2317. 27246235 10.1093/jac/dkw173 PMC4954929

[B31] Poirel L. Jayol A. Nordmann P. (2017). Polymyxins: antibacterial activity, susceptibility testing, and resistance mechanisms encoded by plasmids and chromosomes. *Clin. Microbiol. Rev.* 30 557–596. 10.1128/CMR.00064-16 28275006 PMC5355641

[B32] Pournaras S. Poulou A. Dafopoulou K. Chabane Y. N. Kristo I. Makris D. (2014). Growth retardation, reduced invasiveness, and impaired colistin-mediated cell death associated with colistin resistance development in *Acinetobacter baumannii*. *Antimicrob. Agents Chemother.* 58 828–832. 10.1128/AAC.01439-13 24247145 PMC3910856

[B33] Sato T. Shiraishi T. Hiyama Y. Honda H. Shinagawa M. Usui M. (2018). Contribution of novel amino acid alterations in PmrA or PmrB to colistin resistance in *mcr*-negative *Escherichia coli* clinical isolates, including major multidrug-resistant lineages O25b:H4-ST131-*H*30Rx and Non-x. *Antimicrob. Agents Chemother.* 62:e864-18. 10.1128/AAC.00864-18 29914952 PMC6125499

[B34] Siu L. K. Fung C. P. Chang F. Y. Lee N. Yeh K. M. Koh T. H. (2011). Molecular typing and virulence analysis of serotype K1 *Klebsiella pneumoniae* strains isolated from liver abscess patients and stool samples from noninfectious subjects in Hong Kong, Singapore, and Taiwan. *J. Clin. Microbiol.* 49 3761–3765. 10.1128/JCM.00977-11 21900521 PMC3209116

[B35] Sun J. Zhang H. Liu Y. H. Feng Y. (2018). Towards understanding MCR-like colistin resistance. *Trends Microbiol.* 26 794–808. 10.1016/j.tim.2018.02.006 29525421

[B36] Tietgen M. Semmler T. Riedel-Christ S. Kempf V. A. J. Molinaro A. Ewers C. (2018). Impact of the colistin resistance gene *mcr-1* on bacterial host. *Int. J. Antimicrob. Agents* 51 554–561. 29180279 10.1016/j.ijantimicag.2017.11.011

[B37] Wand M. E. Bock L. J. Bonney L. C. Sutton J. M. (2015). Retention of virulence following adaptation to colistin in *Acinetobacter baumannii* reflects the mechanism of resistance. *J. Antimicrob. Chemother.* 70 2209–2216. 25904728 10.1093/jac/dkv097

[B38] Wang R. Liu Y. Zhang Q. Jin L. Wang Q. Zhangt Y. (2018). The prevalence of colistin resistance in *Escherichia coli* and *Klebsiella pneumoniae* isolated from food animals in China: coexistence of *mcr-1* and *bla*NDM with low fitness cost. *Int. J. Antimicrob. Agents* 51 739–744. 10.1016/j.ijantimicag.2018.01.023 29409993

[B39] Wirth T. Falush D. Lan R. Colles F. Mensa P. Wieler L. H. (2006). Sex and virulence in *Escherichia coli*: an evolutionary perspective. *Mol. Microbiol.* 60 1136–1151. 10.1111/j.1365-2958.2006.05172.x 16689791 PMC1557465

[B40] Wu R. Yi L. X. Yu L. F. Wang J. Liu Y. Chen X. (2018). Fitness advantage of *mcr-1*-bearing IncI2 and IncX4 plasmids in vitro. *Front. Microbiol.* 9:331 10.3389/fmicb.2017.0331 PMC5835064 29535696

[B41] Xu Y. Wei W. Lei S. Lin J. Srinivas S. Feng Y. (2018). An evolutionarily conserved mechanism for intrinsic and transferable polymyxin resistance. *mBio* 9:e2317-17. 10.1128/mBio.02317-17 PMC5893884 29636432

[B42] Zhang Y. Liao K. Gao H. Wang Q. Wang X. Li H. (2017). Decreased fitness and virulence in ST10 *Escherichia coli* harboring *bla*NDM-5 and *mcr-1* against a ST4981 strain with *bla*NDM-5. *Front. Cell Infect. Microbiol.* 7:242 10.3389/fmicb.2017.0242 PMC5463033 28642846

