# Supplementary material for: Comparison of Fitness Cost and Virulence in Chromosome- and Plasmid-Mediated Colistin-Resistant Escherichia coli
Source: Front Microbiol. 2020 May 13;11:798. doi: 10.3389/fmicb.2020.00798 (PMC7238749; doi:10.3389/fmicb.2020.00798)

**Supplementary Table S1.** Primers used for amplifying and sequencing *basRS*, *phoPQ* and *eptA*

| Gene | Primer | Sequence (5' - 3') | Amplicon size (bp) | Gene length (bp) | Amino acid length (aa) |
|---|---|---|---|---|---|
| *basR* | Eco-basR_F | CGCAAACGCAACACTATTCA | 975 | 669 | 223 |
| | Eco-basR_R | ATGATGTGGCGATCGTTGTT | | | |
| *basS* | Eco-basS_F | GACAATGAACCCTCGACCAA | 1,605 | 1,092 | 364 |
| | Eco-basS_F1 | GTGCGTTTGCATCTGGAACT | | | |
| | Eco-basS_R | CGGCGTAACCATGAAAGAGA | | | |
| *phoP* | Eco-phoP_F | CCCCCATAACCACATAATCG | 794 | 672 | 224 |
| | Eco-phoP_R | CGTTGCCAACAGAAAACGTA | | | |
| *phoQ* | Eco-phoQ_F | CGCGTTCGAATACACCATTA | 1,774 | 1,461 | 487 |
| | Eco-phoQ_F1 | TGCTGAGTGGAGATCATTCG | | | |
| | Eco-phoQ_R | CAAGAAAATCGGGCCAGTTA | | | |
| *eptA* | Eco-eptA_F | CCAGAATCAGTCCCTGCAAT | 1,797 | 1,644 | 548 |
| | Eco-eptA_R | TCCCCTTAATCCAGCAAACA | | | |

**Supplementary Table S2.** Primers for qRT-PCR

| Gene | Primer | Sequence (5' - 3') | Amplicon size (bp) |
|---|---|---|---|
| *rrsH* | rrsH_F | GAATGCCACGGTGAATACGTT | 64 |
| | rrsH_R | ACCCACTCCCATGGTGTGA | |
| *basR* | basR_F | ATTGCAGGGACTGATTCTGG | 134 |
| | basR_R | CGTCGGGTAACCCTAAATCC | |
| *phoP* | phoP_F | GCAGAAGATGCCAAAGAAGC | 143 |
| | phoP_R | ACCAGAATCGGCAGTGAAAC | |
| *eptA* | eptA_F | TCCCTAATACCGCATCTTGC | 124 |
| | eptA_R | ATGATATCCAGCACGCCTTC | |

**Supplementary Table S3.** Antimicrobial resistance profile of the *E. coli* strains.

| *E. coli* strain | Minimum Inhibitory Concentration (mg/L)[a, b, c] | | | | | | | | | |
|---|---|---|---|---|---|---|---|---|---|---|
| | CL | IMI | AMK | CPM | CRO | CIP | TET | RIF | P/T | A/S |
| E015 | 0.25 (S) | 0.125 (S) | 16 (I) | 64 (R) | >64 (R) | 16 (R) | 64 (R) | 32 | 2/4 (S) | 32/16 (R) |
| E139 | 0.25 (S) | 0.125 (S) | 8 (S) | >64 (R) | >64 (R) | 64 (R) | 4 (S) | 16 | 64/4 (I) | >64/32 (R) |
| E154 | 0.25 (S) | 0.125 (S) | 4 (S) | >64 (R) | >64 (R) | 32 (R) | 4 (S) | 32 | 4/4 (S) | >64/32 (R) |
| E015R | 64 (R) | 0.125 (S) | 2 (S) | >64 (R) | >64 (R) | 16 (R) | >64 (R) | 32 | 4/4 (S) | 64/32 (R) |
| E139R | >64 (R) | 0.125 (S) | 2 (S) | 0.25 (S) | 0.063 (S) | >64 | 2 (S) | 32 | 4/4 (S) | 64/32 (R) |
| E154R | 64 (R) | 0.125 (S) | 2 (S) | 0.125 (S) | 0.063 (S) | 0.063 (S) | 64 (R) | 8 | 2/4 (S) | 64/32 (R) |
| QIA18 | 8 (R) | 0.125 (S) | 2 (S) | 0.125 (S) | 0.063 (S) | 64 (R) | >64 (R) | 64 | 4/4 (S) | 64/32 (R) |
| QIA24 | 8 (R) | 0.063 (S) | 2 (S) | 0.125 (S) | 0.063 (S) | 0.063 (S) | >64 (R) | 64 | 8/4 (S) | 64/32 (R) |
| QIA32 | 8 (R) | 0.125 (S) | 1 (S) | 0.125 (S) | 0.063 (S) | 0.25 (S) | 64 (R) | 16 | 2/4 (S) | 64/32 (R) |
| QIA33 | 16 (R) | 0.063 (S) | 2 (S) | 0.125 (S) | 0.063 (S) | 0.25 (S) | 32 (R) | 16 | 2/4 (S) | 32/16 (R) |
| EC006 | 8 (R) | 0.125 (S) | 2 (S) | >64 (R) | >64 (R) | 16 (R) | >64 (R) | 64 | 8/4 (S) | 64/32 (R) |
| EC019 | 8 (R) | 0.125 (S) | 4 (S) | >64 (R) | >64 (R) | 64 (R) | 2 (S) | 32 | 8/4 (S) | 64/32 (R) |
| EC111 | 16 (R) | 0.125 (S) | 2 (S) | >64 (R) | >64 (R) | 32 (R) | 2 (S) | 32 | >256/4 (R) | >64/32 (R) |
| $J53_{pEC006}$ | 4 (R) | 0.25 (S) | 2 (S) | 0.25 (S) | 0.063 (S) | 0.063 (S) | 2 (S) | 32 | 4/4 (S) | 32/16 (R) |
| $J53_{pEC019}$ | 4 (R) | 0.125 (S) | 2 (S) | 0.125 (S) | 0.063 (S) | 0.063 (S) | 2 (S) | 16 | 8/4 (S) | 32/16 (R) |
| $J53_{pEC111}$ | 4 (R) | 0.125 (S) | 2 (S) | 0.125 (S) | 0.063 (S) | 0.063 (S) | 2 (S) | 32 | 8/4 (S) | 32/16 (R) |
| MG1655 | 0.25 (S) | - | - | - | - | - | - | | - | - |
| J53 | 0.25 (S) | - | - | - | - | - | - | | - | - |

[a] CL, colistin; PB, polymyxin B; IMI, imipenem; AMK, amikacin; CPM, cefepime; CRO, ceftriaxone; CIP, ciprofloxacin; TET, tetracycline; RIF, rifampicin; P/T, piperacillin-tazobactam; A/S, ampicillin-sulbactam

[b] S, susceptible; I, intermediate-resistant; R, resistant

[c] There is no CLSI breakpoint for rifampicin

**Supplementary Figure S1. The results of MALDI-TOF mass spectrometry.** (A) Isogenic strains E015 and E015R, (B) a parental *E. coli* J53 and a transconjugant $J53_{pEC019}$, and (C) WT *mcr-1*-positive strains EC006, EC019, and EC111. Bis-phosphorylated and hexa-acylated lipid A peak (1797.2 EM) was identified in all isolates. An addition of PEtN to the hexa-acylated lipid A (1920 EM) was detected from strains displaying colistin resistance.

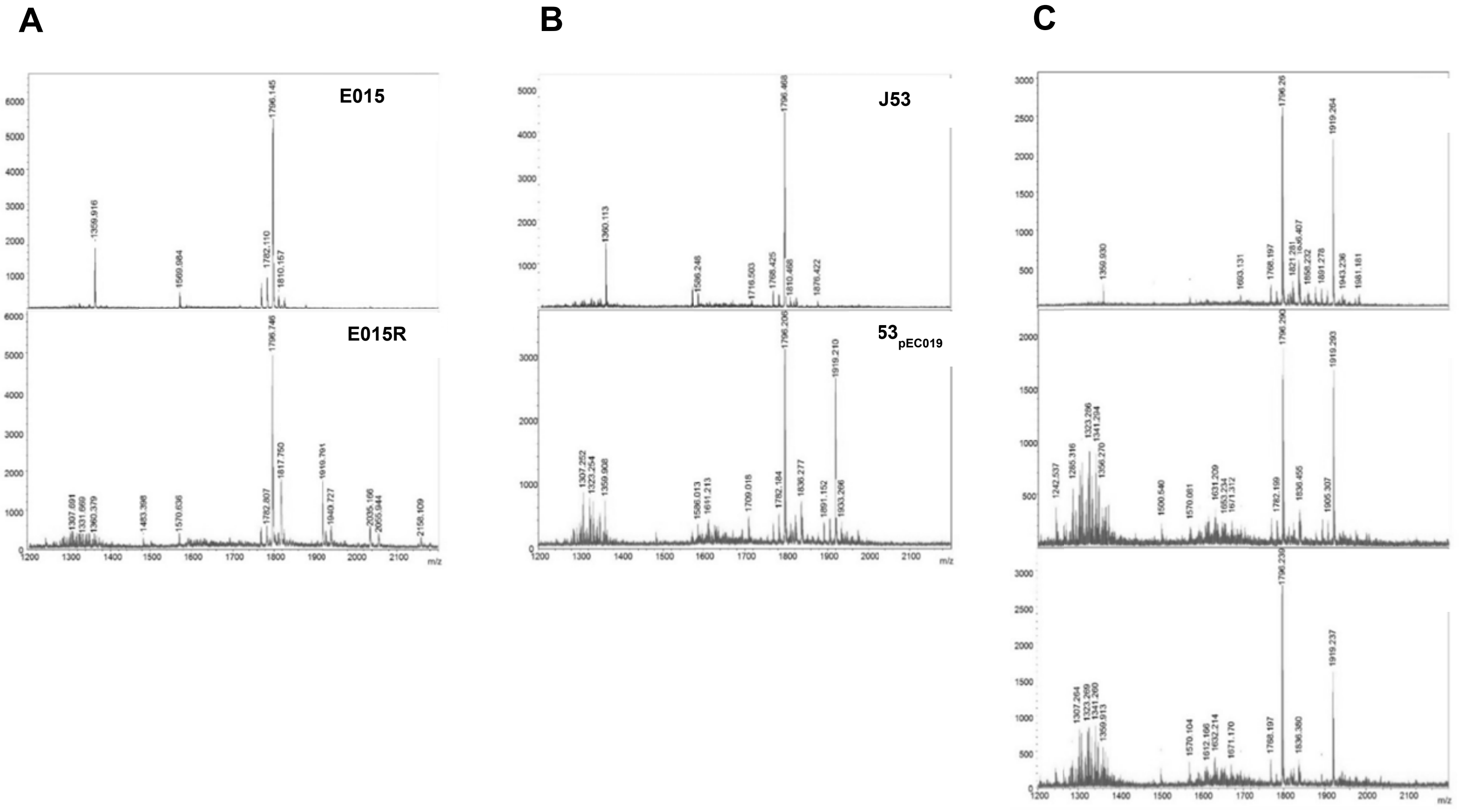

Supplement: Supplementary file 1 [file Data_Sheet_1.pdf]
